# Identification of a novel senescence-associated signature to predict biochemical recurrence and immune microenvironment for prostate cancer

**DOI:** 10.3389/fimmu.2023.1126902

**Published:** 2023-02-20

**Authors:** Chenglin Han, Yuxuan Deng, Bin Yang, Peng Hu, Bintao Hu, Tao Wang, Jihong Liu, Qidong Xia, Xiaming Liu

**Affiliations:** Department of Urology, Tongji Hospital, Tongji Medical College, Huazhong University of Science and Technology, Wuhan, China

**Keywords:** prostate cancer, cell senescence, biochemistry recurrence, prognostic signature, tumor immunity

## Abstract

**Background:**

Prostate cancer (PCa) is an age-associated malignancy with high morbidity and mortality rate, posing a severe threat to public health. Cellular senescence, a specialized cell cycle arrest form, results in the secretion of various inflammatory mediators. In recent studies, senescence has shown an essential role in tumorigenesis and tumor development, yet the extensive effects of senescence in PCa have not been systematically investigated. Here, we aimed to develop a feasible senescence-associated prognosis model for early identification and appropriate management in patients with PCa.

**Method:**

The RNA sequence results and clinical information available from The Cancer Genome Atlas (TCGA) and a list of experimentally validated senescence-related genes (SRGs) from the CellAge database were first obtained. Then, a senescence-risk signature related with prognosis was constructed using univariate Cox and LASSO regression analysis. We calculated the risk score of each patient and divided them into high-risk and low-risk groups in terms of the median value. Furthermore, two datasets (GSE70770 and GSE46602) were used to assess the effects of the risk model. A nomogram was built by integrating the risk score and clinical characteristics, which was further verified using ROC curves and calibrations. Finally, we compared the differences in the tumor microenvironment (TME) landscape, drug susceptibility, and the functional enrichment among the different risk groups.

**Results:**

We established a unique prognostic signature in PCa patients based on eight SRGs, including CENPA, ADCK5, FOXM1, TFAP4, MAPK, LGALS3, BAG3, and NOX4, and validated well prognosis-predictive power in independent datasets. The risk model was associated with age and TNM staging, and the calibration chart presented a high consistency in nomogram prediction. Additionally, the prognostic signature could serve as an independent prediction factor due to its high accuracy. Notably, we found that the risk score was positively associated with tumor mutation burden (TMB) and immune checkpoint, whereas negatively correlated with tumor immune dysfunction and exclusion (TIDE), suggesting that these patients with risk scores were more sensitive to immunotherapy. Drug susceptibility analysis revealed differences in the responses to general drugs (docetaxel, cyclophosphamide, 5-Fluorouracil, cisplatin, paclitaxel, and vincristine) were yielded between the two risk groups.

**Conclusion:**

Identifying the SRG-score signature may become a promising method for predicting the prognosis of patients with PCa and tailoring appropriate treatment strategies.

## Introduction

Prostate cancer (PCa) is a highly prevalent malignancy in men worldwide, and its incidence still exhibits a steady growth because of the popularity of serum prostate-specific antigen (PSA) screening (1). Although conventional PSA test contributes to early intervention before metastasis, overdiagnosis, and overtreatment are inevitable in therapy due to its poor specificity. Clinicians may perform unnecessary biopsies or immoderate over-treatment of low-risk PCa patients. Radical prostatectomy (RP) and radiotherapy are considered standard clinical management strategies for patients with localized prostate neoplasm. Unsatisfactorily, about one-third of patients still encounter biochemical recurrence (BCR) during follow-up, indicating a risk of underlying clinical metastases and poor prognosis (2, 3). Given the high dependency of PCa cells on androgen for proliferation and survival, androgen deprivation therapy (ADT) is initially capable of providing oncological control and symptomatic improvement in most patients; however, these patients ultimately relapse and develop into advanced castration‐resistant prostate cancer (CRPC) within two years, and the five-year overall survival rate is not optimistic. As each biological biomarker has its own limitations, thus it is of great significance to explore reliable molecular markers and prognosis models that contribute to early diagnosis and inform decision-making in the era of precision medicine.

Aging is a gradual decline of an organism over time. Emerging evidence has demonstrated a subtle connection between PCa and aging phenotype, particularly cellular senescence characterized by a typically irreversible growth arrest and morphology alteration and senescence-associated secretory phenotype (SASP) (4). SASP mainly refers to the secretion of various bioactive molecules, including pro-inflammatory cytokines, growth factors, and metalloproteases. During the aging process, cellular senescence is triggered by multiple intrinsically and extrinsically detrimental stresses, such as oxidative stress, DNA damage, telomere shorting, and inappropriate activation of oncogenes (5). Interestingly, cellular senescence exhibits dual roles in the initiation and growth of tumors, which partially results in intratumor heterogeneity to a certain extent (6). In the past decades, senescence was defined as an adaptive response of cells against unfavorable conditions. In the context of cancer, senescence-mediated life stagnation is a critical intrinsic mechanism of antitumor defense since (pre)neoplastic cells can be prevented from proliferation and progression (7). This concept has recently been queried by conflicting evidence showing that non-malignant and malignant cells with lastingly persistent senescence can acquire carcinogenic properties.

Indeed, senescent cells have been demonstrated to exist in the murine and human tumor microenvironment (8). Though mitotically inactive, senescent cells are metabolically active. They undergo chromosomal aberrations and generate a “fertile” microenvironment through SASP release, ultimately leading to their malignant transformation. Fibroblasts account for a large component within tissues, therefore the pro-tumoral senescent microenvironment remodeling is inextricably related to a shift in fibroblast behavior (9). The senescent fibroblasts significantly increased the number of immunosuppressive myeloid-derived suppressor cells (MDSCs) and Treg cells, mainly through IL-6 secretion, while averting PD-L1-mediated immunosuppression by releasing amphiregulin (10, 11). Regardless of their specific mechanisms, anticancer chemotherapeutics inevitably induced a senescent phenotype transformation in stromal fibroblasts and their paracrine secretion activity, sustaining the clonogenic and invasive potential of PCa cells (12). Remarkably, the alteration of inflammatory cytokines in senescent dendritic cells (DCs) and effector T cells was not sufficient to achieve immune-mediated clearance of tumor cells. Until now, the extent to which cellular senescence facilitates PCa is still clearly clarified.

Because of the tumor heterogeneity, a thorough understanding of senescence could provide valuable insights into tumor formation and progression. In the study, we explored the expression patterns of cellular senescence-related genes (SRGs) and developed a prognosis-predictive signature based on eight SRGs (CENPA, ADCK5, FOXM1, TFAP4, MAPK, LGALS3, BAG3, and NOX4) using bioinformatic technology. Additionally, a practical senescence-risk algorithm was built to proceed with quantitative risk stratification of patients with PCa. Our study demonstrated that the prognosis of patients with different risk scores was discrepant, and revealed the relationship between the senescence-risk score and clinicopathologic characteristics, as well as the immune microenvironment. It also correlated with TMB, TIDE, and chemotherapeutic sensitivity. Thus, this research would provide valuable biomarkers for PCa diagnosis and monitoring and aid in determining the best patient-specific course of treatment.

## Methods

### Data source

The RNA-seq results and corresponding clinical characteristics in PCa samples were obtained from The Cancer Genome Atlas (TCGA) database and Gene Expression Omnibus (GEO) database. The profiles of SRGs (**Table S1**) were extracted from the CellAge Data Portal. Notably, GSE70770 and GSE46602 were employed as external validation datasets.

### Defining differentially expressed SRGs

Gene expression patterns were log2 transformed using the “edgeR” package, then the differentially expressed genes (DEGs) in normal prostate tissues and PCa tissues were obtained by the R package “limma”. Notably, the filter thresholds were set as follows: |log2 (FC)| > 1 and the false discovery rate (FDR)< 0.05. Volcano Plot and heatmap were conducted with the “pheatmap” R package for visualizing the gene expression differences. Finally, we performed an intersection of SRGs and DEGs and acquired a group of differentially expressed SRGs for further analysis. Following this, we performed univariate Cox regression to screen SRGs with prognostic value. Differential expression SRGs with univariate Cox regression p< 0.05 were regarded as essential SRGs in PCa. Interested in whether protein level expression of these essential SRGs is stable. We systematically investigated the mutation atlas and co-mutation status of these essential SRGs in PCa.

### Construction and evaluation of the prognostic model

Having identified essential SRGs associated with prognosis. Then, we used the least absolute shrinkage and selection operator (LASSO) regression to achieve the final elimination of potential indicators with nonzero coefficients (13). The risk model was eventually established based on the standardized expression levels of these screened variables weighted by their coefficients derived from the LASSO regression analysis. The risk score was calculated using the following formula:


$$risk\;score=\sum_{i=1}^{n}ki*Xi$$

Here, k and X represent their relative expression levels and regression coefficients. To avoid extreme values and equally reflect on the senescence degrees, we divided patients into high- and low-risk subgroups with the cut-off of the median risk value. A TCGA-PCa internal cohort and two GEO external cohorts (GSE70770 and GSE46602) were applied to check the validity of the predictive model. We investigated the relevance of risk scores to clinical variables by wilcoxon test, and respectively estimated their independent prognostic values through the univariate/multivariate Cox regression. ROC curves were plotted to evaluate the predictive performance of different clinical pathological characteristics. We developed a nomogram for predicting non-BCR probability on account of the risk score and other clinical factors, which was further evaluated by calibration curves.

### Analysis of immune landscapes

The tumor mutational burden (TMB) data were calculated from the TCGA_PRAD cohort. The association between risk score and TMB was then analyzed. We also compared the differences in frequencies of the top mutant 20 genes in the two senescence-risk groups. The Kaplan-Meier (KM) method was applied to compare the differences in BCR-free survival between patients with multiple modes of risk and TMB. We estimated the relative abundance of immune and stromal cells of each patient from the TCGA database by applying different algorithms (TIMER, CIBERSORT, QUANTISEQ, CIBERSORT-ABS, MCPCOUNTER, XCELL, and EPIC). In addition, correlation coefficients were calculated to determine the relationship between risk score and infiltrated immune cells. Moreover, we explored the expression levels of a panel of essential immune checkpoint targets in high- and low-risk groups. The tumor immune dysfunction and exclusion (TIDE) score was employed to reflect the immune evasion of tumor cells and their response to immune checkpoint inhibitors (ICIs). An association analysis between risk score and TIDE value was performed, and the differences in TIDE value among the two subgroups were examined using the Wilcoxon test.

### Drug sensitivity prediction

The ProPhetic algorithm was used to predict the response to common therapeutic agents, and the half maximal inhibitory concentration (IC50) was then compared to investigate the drug sensitivity to conventional chemotherapy among high- and low-risk patients through “pRRophetic” R package.

### Gene set enrichment analysis (GSEA) and gene set variation analysis (GSVA)

The Gene Ontology (GO) and Kyoto Encyclopedia of Genes and Genome (KEGG) analyses were carried out on the screened candidate genes using the “ClusterProfiler” R package, and corresponding GSVA analysis were carried out by R package “GSVA” to determine whether the risk score was correlated with the senescence levels in PCa patients.

### Protein expression patterns verification of modeling genes

From the Human Protein Atlas (HPA) database (https://www.proteinatlas.org/), the immunohistochemical results of the final genes enrolled in the predictive model were further obtained to support their differential expression status between PCa and normal tissue in protein level.

## Results

### Screening of differentially expressed prognostic SRGs

**Figure 1:**
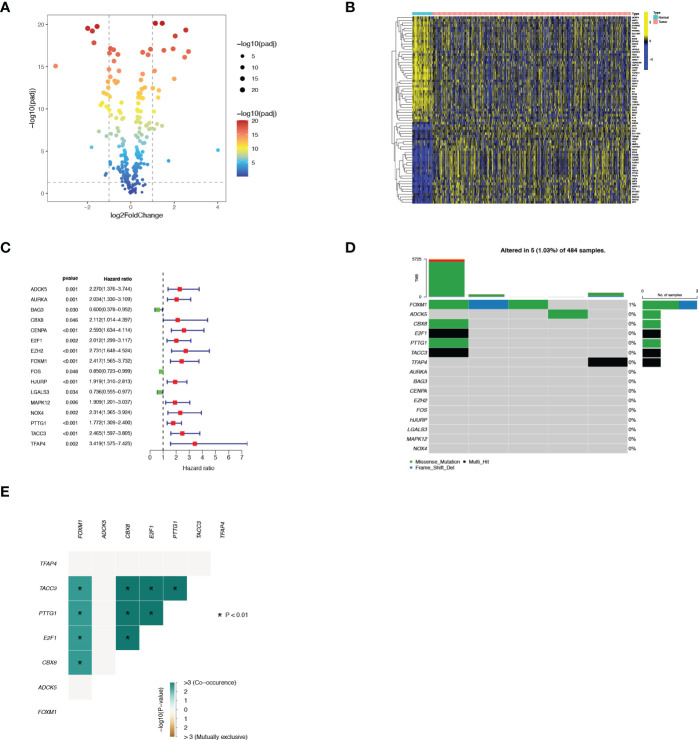
Identification of 16 vital differentially expressed SRGs in PRAD. **(A)** Volcano map of differentially expressed SRGs. **(B)** Heatmap of differentially expressed SRGs. **(C)** Forest plot displayed 16 prognosis-associated genes identified by univariate Cox regression. **(D)** The mutation atlas of these essential SRGs. **(E)** Co-mutation status of these essential SRGs.

A total of 663 PCa patients obtained from 3 independent cohorts were included in this study, and their basic characteristics were summarized in **Table 1**. Firstly, to systematically delineate the impact of senescence in prostate tumors, we first extracted the SRGs from the CellAge database and compared their expression in tumor tissues versus adjacent tissues from the TCGA database. With a cut-off |log2FC|>1 and FDR<0.05, the profiles of differentially expressed SRGs were displayed in volcano and heatmap plots (**Figures 1A, B**), which revealed 40 down-regulated genes and 31 up-regulated genes (**Table S2**). Of these, 13 risky genes (hazard ratio, HR > 1) and 3 protective genes were finally sorted out as biochemical recurrence (BCR)-associated factors through univariate Cox regression analyses, as illustrated in **Figure 1C**. Furthermore, the mutation probability of these 16 genes was only 1.03% in 484 prostate tumor samples, yet several genes could be mutated simultaneously (**Figures 1D, E**). These findings demonstrated that the functions of preliminarily selected SRGs were highly stable and connected.

**Table 1:** The basic characteristics of patients included in the three cohorts.

	Overall	GSE46602	GSE70770	TCGA	p
n	663	36	203	424	
Age (%)					<0.001
41	2 (0.3)	0 (0.0)	1 (0.5)	1 (0.2)	
42	1 (0.2)	0 (0.0)	1 (0.5)	0 (0.0)	
43	1 (0.2)	0 (0.0)	0 (0.0)	1 (0.2)	
44	3 (0.5)	0 (0.0)	1 (0.5)	2 (0.5)	
46	6 (0.9)	1 (2.8)	0 (0.0)	5 (1.2)	
47	6 (0.9)	0 (0.0)	1 (0.5)	5 (1.2)	
48	6 (0.9)	0 (0.0)	2 (1.0)	4 (0.9)	
49	6 (0.9)	0 (0.0)	0 (0.0)	6 (1.4)	
50	9 (1.4)	0 (0.0)	3 (1.5)	6 (1.4)	
51	11 (1.7)	0 (0.0)	2 (1.0)	9 (2.1)	
52	11 (1.7)	1 (2.8)	2 (1.0)	8 (1.9)	
53	16 (2.4)	1 (2.8)	1 (0.5)	14 (3.3)	
54	18 (2.7)	0 (0.0)	5 (2.5)	13 (3.1)	
55	25 (3.8)	1 (2.8)	6 (3.0)	18 (4.2)	
56	23 (3.5)	0 (0.0)	5 (2.5)	18 (4.2)	
57	35 (5.3)	3 (8.3)	5 (2.5)	27 (6.4)	
58	26 (3.9)	3 (8.3)	4 (2.0)	19 (4.5)	
59	26 (3.9)	4 (11.1)	4 (2.0)	18 (4.2)	
60	22 (3.3)	1 (2.8)	5 (2.5)	16 (3.8)	
61	32 (4.8)	1 (2.8)	7 (3.4)	24 (5.7)	
62	33 (5.0)	1 (2.8)	11 (5.4)	21 (5.0)	
63	38 (5.7)	4 (11.1)	9 (4.4)	25 (5.9)	
64	32 (4.8)	1 (2.8)	6 (3.0)	25 (5.9)	
65	26 (3.9)	1 (2.8)	5 (2.5)	20 (4.7)	
66	33 (5.0)	0 (0.0)	4 (2.0)	29 (6.8)	
67	29 (4.4)	3 (8.3)	8 (3.9)	18 (4.2)	
68	29 (4.4)	6 (16.7)	1 (0.5)	22 (5.2)	
69	19 (2.9)	3 (8.3)	5 (2.5)	11 (2.6)	
70	12 (1.8)	0 (0.0)	2 (1.0)	10 (2.4)	
71	10 (1.5)	1 (2.8)	1 (0.5)	8 (1.9)	
72	11 (1.7)	0 (0.0)	2 (1.0)	9 (2.1)	
73	6 (0.9)	0 (0.0)	2 (1.0)	4 (0.9)	
74	2 (0.3)	0 (0.0)	0 (0.0)	2 (0.5)	
75	3 (0.5)	0 (0.0)	0 (0.0)	3 (0.7)	
76	1 (0.2)	0 (0.0)	0 (0.0)	1 (0.2)	
77	1 (0.2)	0 (0.0)	0 (0.0)	1 (0.2)	
78	1 (0.2)	0 (0.0)	0 (0.0)	1 (0.2)	
unknow	92 (13.9)	0 (0.0)	92 (45.3)	0 (0.0)	
T (%)					0.316
T1-2	253 (38.2)	19 (52.8)	81 (39.9)	153 (36.1)	
T3-4	402 (60.6)	17 (47.2)	119 (58.6)	266 (62.7)	
unknow	8 (1.2)	0 (0.0)	3 (1.5)	5 (1.2)	
BCR = BCR/Non-BCR (%)	142/521 (21.4/78.6)	22/14 (61.1/38.9)	64/139 (31.5/68.5)	56/368 (13.2/86.8)	<0.001
ADCK5 (mean (SD))	3.86 (0.46)	3.92 (0.42)	3.85 (0.42)	3.86 (0.47)	0.698
BAG3 (mean (SD))	6.55 (0.62)	6.39 (0.54)	6.49 (0.66)	6.59 (0.61)	0.054
CENPA (mean (SD))	2.76 (0.44)	2.74 (0.42)	2.83 (0.45)	2.72 (0.44)	0.016
FOXM1 (mean (SD))	3.21 (0.46)	3.32 (0.42)	3.25 (0.40)	3.19 (0.49)	0.091
LGALS3 (mean (SD))	6.35 (0.88)	6.04 (0.73)	6.36 (0.93)	6.37 (0.87)	0.095
MAPK12 (mean (SD))	3.52 (0.52)	3.61 (0.47)	3.60 (0.53)	3.48 (0.52)	0.013
NOX4 (mean (SD))	2.82 (0.41)	2.88 (0.44)	2.90 (0.40)	2.78 (0.40)	0.003
TFAP4 (mean (SD))	3.50 (0.31)	3.54 (0.34)	3.54 (0.30)	3.47 (0.31)	0.017
riskScore (median [IQR])	3.81 [3.50, 4.13]	3.97 [3.78, 4.14]	3.89 [3.63, 4.16]	3.75 [3.42, 4.10]	<0.001
Risk = high/low (%)	374/289 (56.4/43.6)	28/8 (77.8/22.2)	134/69 (66.0/34.0)	212/212 (50.0/50.0)	<0.001

### Construction of prognostic model

**Figure 2:**
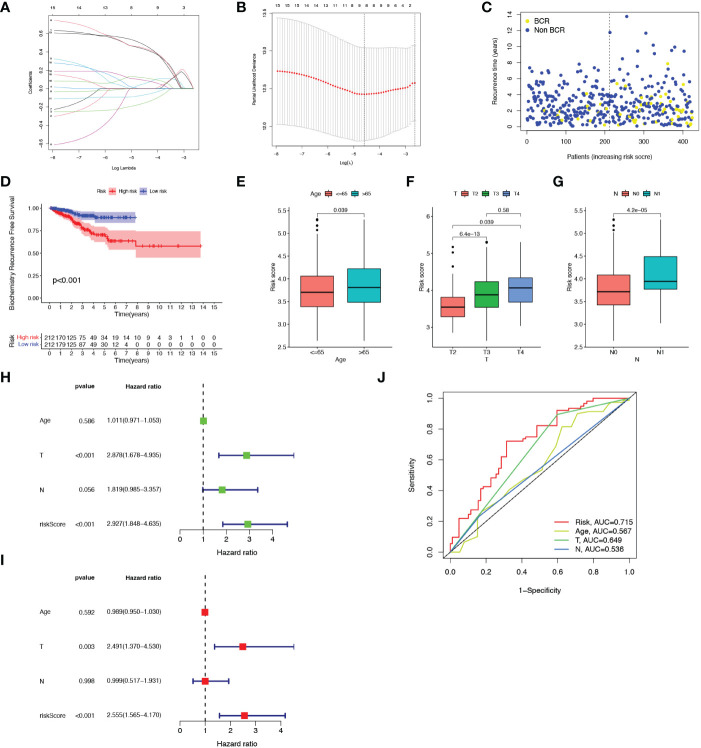
Construction of the prognostic model. **(A, B)** Results of LASSO regression analysis. **(C)** Scatter plot of BCR status of each patient in the TCGA cohort. **(D)** Kaplan-Meier BCR curves between high- and low-risk groups. **(E-G)** Correlation of clinical features (Age, T staging and N staging) with risk score. **(H, I)** Forest plots showed the association between clinicopathological features (including risk score) and prognosis through univariate and multivariate Cox regression analysis. **(J)** ROC curves of prognostic factors.

Thereafter, LASSO regression analysis and tenfold cross-validation was performed to identify more meaningful variables for the senescence-risk signature associated with BCR. The LASSO coefficient profiles were generated against the log(k) sequence and the optimal parameter (**Figures 2A, B**). We eventually determined 8 hub genes and their regression coefficients in the prognostic risk model (**Table 2**). **Figure S1A** showed that they closely interacted with each other.

**Table 2:** The senescence effects of corresponding coefficients of 8 hub genes in this risk model.

Gene	Senescence _effect	Coef
ADCK5	Induces	0.436959694590544
BAG3	Inhibits	-0.164716968019639
CENPA	Inhibits	0.0464710448463931
FOXM1	Inhibits	0.447384503744576
LGALS3	Inhibits	-0.073349813641182
MAPK12	Induces	0.0947576387538311
NOX4	Induces	0.489534901499645
TFAP4	Induces	0.120995001609793

We calculated the risk score of each sample from the TCGA-PRAR cohort according to the formula mentioned above, and then separated all patients into high-risk (n=212) and low-risk (n=212) subgroups with the median value of 3.75 (**Figure S1B**). As shown in **Figures 2C, D**, patients with high-senescence scores were more likely to develop BCR in the advanced stage when compared to those in the low group. In addition, the high-risk and low-risk clusters could be distinguished and visualized in the PCA plot according to the risk score model, whereas it was impossible to separate the two subsets using all SRGs (**Figures S1C, D**).

**Figure S1E** depicted the relationship of the expression of 8 candidate genes with clinical attributes, including T staging, N staging, and age. There were statistical differences in the senescence risk score among age ((≤65 and >65), T-staging (T2, T3, T4), and N-staging (N0, N1), as elucidated in **Figures 2E–G**. The risk score raised as the pathological staging increased, which might represent a worse clinical outcome. Next, univariate and multivariate Cox regression analysis were applied to explore the prognosis-predictive value of risk score and above clinical parameters. As presented in **Figures 2H, I**, both T staging and risk score were independent prognostic indicators for PCa. The ROC curve demonstrated that the predictive model exhibited high sensitivity and specificity, with an area under the curve (AUC) value of 0.715 (**Figure 2J**). Therefore, we speculated that the senescence-risk signature was acceptable.

### Validation of the risk score in the test set

**Figure 3:**
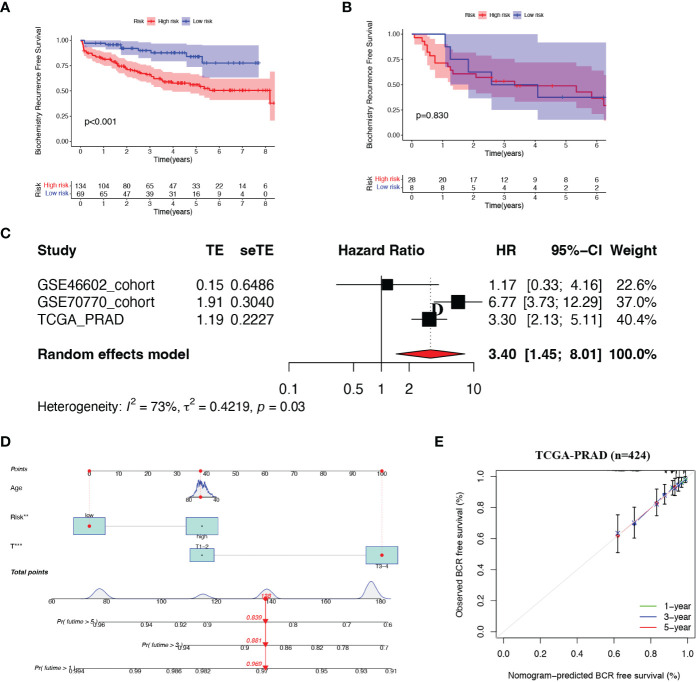
Validation of the risk model **(A, B)** Kaplan-Meier curves of patients in two risk groups. **(C)** Meta-analysis of three cohorts. **(D)** Nomogram was constructed based on Age, risk score and T staging. **(E)** Calibration curves of 1-. 3- and 5- year BCR-free survival.

To further verify this prognostic index’s reliability, we selected two datasets (GSE70770 and GSE46602) as validation tests. Based on the same cut-off value, patients in two cohorts were subdivided into high- and low- risk subsets, respectively (**Figures S2A, B**). There was a negative correlation between the risk score and BCR in PCa patients in the GSE70770 dataset. Nevertheless, no statistically significant difference in BCR was observed between the two groups in the GSE46602 dataset, which may be attributed to the small sample size (**Figures S2C, D**). We respectively evaluated the BCR-free survival status of each sample in the two cohorts and obtained a similar yield described before (**Figures 3A, B**).

Next, we combined all patients from the three cohorts and implemented a meta-analysis (**Figure 3C**). The result indicated that the 8-gene prognostic model was in good validity (HR = 3.40, 95% CI = 1.45–8.01, P = 0.03). Integrating the risk score and two clinical parameters (age and T-staging), we plotted a quantitative algorithm that predicted the percent weight of BCR status in PCa patients (**Figure 3D**). Herein, we randomly plugged a patient into the prognostic nomogram and calculated his non-BCR probability as 0.969, 0.881 and 0.839 at 1, 3 and 5 years, respectively. We established corresponding calibration curves, which showed an excellent consistency between predicted and actual BCR probabilities at 1, 3 and 5 years (**Figure 3E**). Clearly, these findings demonstrated that the nomogram had robust prognostic accuracy.

### Tumor mutation burden analysis

**Figure 4:**
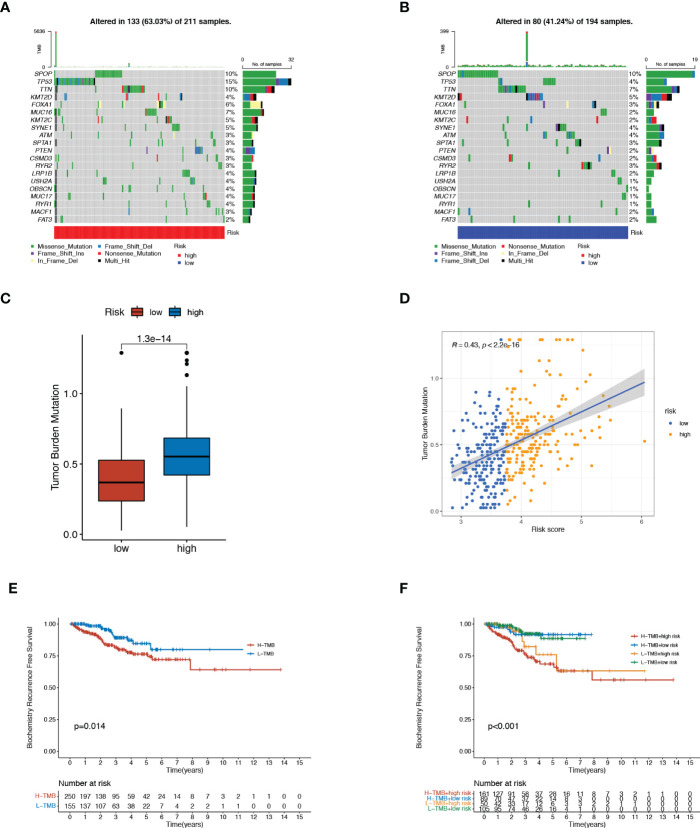
Tumor mutation burden (TMB) analysis. **(A, B)** Mutation spectrum in top 20 genes of high- and low-risk patients. **(C)** Comparation of TMB between the two risk groups. **(D)** Correlation of TMB with risk scores. **(E)** BCR-free survival analysis for high- and low-risk patients. **(F)** BCR-free survival analysis for four groups with different patterns with TMB and risk scores. .

Considering that genetic alterations involved oncogenesis and tumor progression, we drew the mutation spectrum of patients with low-risk and high-risk scores from the TCGA database, respectively (**Figures 4A, B**). Somatic variants analysis displayed the top twenty mutated genes, including SPOP, TP53, and PTEN, consistent with the previously reported conclusions. Among of them, the mutation frequency of the well-known TP53 remarkably increased from 4% to 15%, accompanied by the 2-fold proportion of PTEN mutation in the high-risk group. A significant positive correlation also existed between TMB and the risk score, as illustrated in **Figures 4C, D**. The BCR curve manifested that both TMB and high-risk scores contributed to worse clinical outcomes of PCa (**Figures 4E, F**), which was in line with our expectations.

### Immune microenvironment analysis

**Figure 5:**
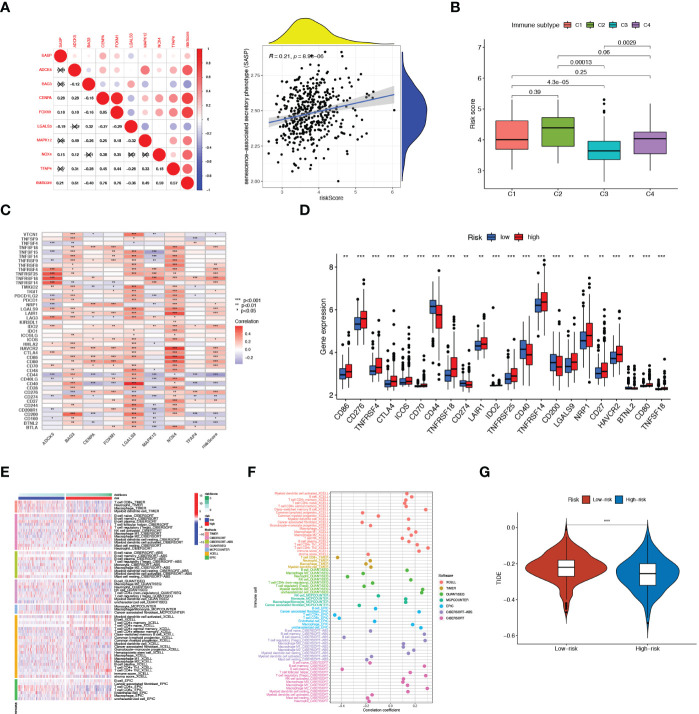
Immune Microenvironment Analysis. **(A)** Correlation of SASP with 8 SRGs as well as risk score As. **(B)** Relationship between risk score and immune subtype. **(C)** Association between immune checkpoints and 8 SRGs. **(D)** The expression levels of immune checkpoints in high- and low-risk patients. **(E)** The distribution alteration of immune-related cells between the two risk groups. **(F)** Correlation of risk scores and immune cell infiltration. **(G)** Comparison of TIDE between high- and low-risk groups. **p < 0.01; ***p < 0.001.

We next assessed the relationship between 8 SRGs as well as senescence scores and senescence-associated secretory phenotype (SASP) activity (**Figure 5A**). Patients with the immune-inflammatory subtype (C3) were considered to have a favorable prognosis (**Figure 5B**), whereas the risk score was negatively associated with the C3 immune subtype. Next, we explored the relationship between the risk score with immune checkpoints (ICPs) to predict immunotherapy benefits. As shown in **Figures 5C, D**, the expression levels of most ICPs, such as CTLA4, CD86, and NRP1, were significantly up-regulated in the high-risk group, and exhibited a positive correlation with the risk score. Next, we employed the seven algorithms to analyze immune microenvironment characteristics (**Figures 5E, F**). We noticed that the risk score could evaluate the distribution differences of immune cell subsets in the prostate tumor tissue. Specifically, the ratio of CD4^+^ and CD8^+^ T cells decreased significantly in high-risk patients, yet with an apparent increase in M2 macrophages, T-cell regulatory (Tregs), and other harmful immune cells.

To determine the best patient-personalized management in the clinical setting, we compared the differences in sensitivity toward chemotherapeutic agents between the two clusters. The TIDE score was applied to assess immunotherapy efficiency, where a lower TIDE value meant a better response to immunotherapy. Patients universally acquired lower TIDE scores in the high-risk group, representing their more sensitivity to immunotherapy (**Figure 5G**).

### The interaction of risk score and chemotherapy sensitivity

**Figure 6:**
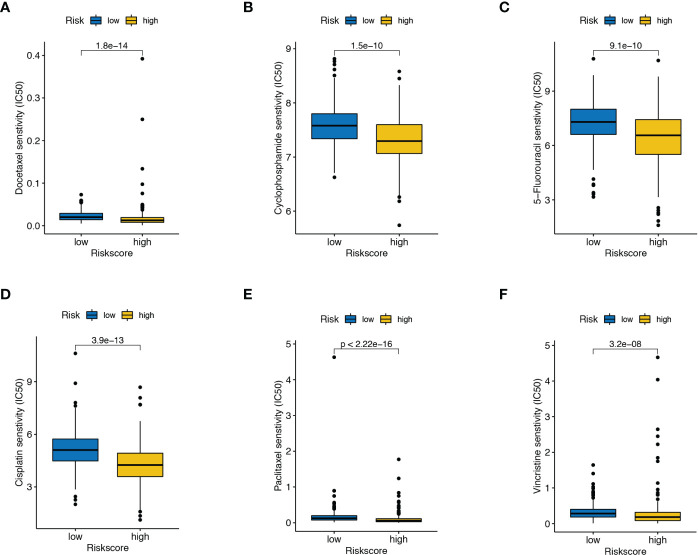
Drug susceptibility analysis. The differences in the response to **(A)** docetaxel, **(B)** cyclophosphamide, **(C)** 5-Fluorouracil, **(D)** cisplatin, **(E)** paclitaxel, and **(F)** vincristine between high- and low-risk score patients.

IC50 referred to the half inhibitory concentration, indicating that the lower the IC50, the more sensitive patients were to therapeutic agents. From the boxplots, we observed that patients with high-risk scores exhibited stronger sensitivity toward most chemotherapeutic drugs, including docetaxel, cyclophosphamide, 5-Fluorouracil, cisplatin, paclitaxel, and vincristine (**Figures 6A–F**). Altogether, these results hint that the combination of ADT and these drugs could improve the antitumor therapeutic potential for such patients with high-risk scores.

### Functional enrichment analysis

**Figure 7:**
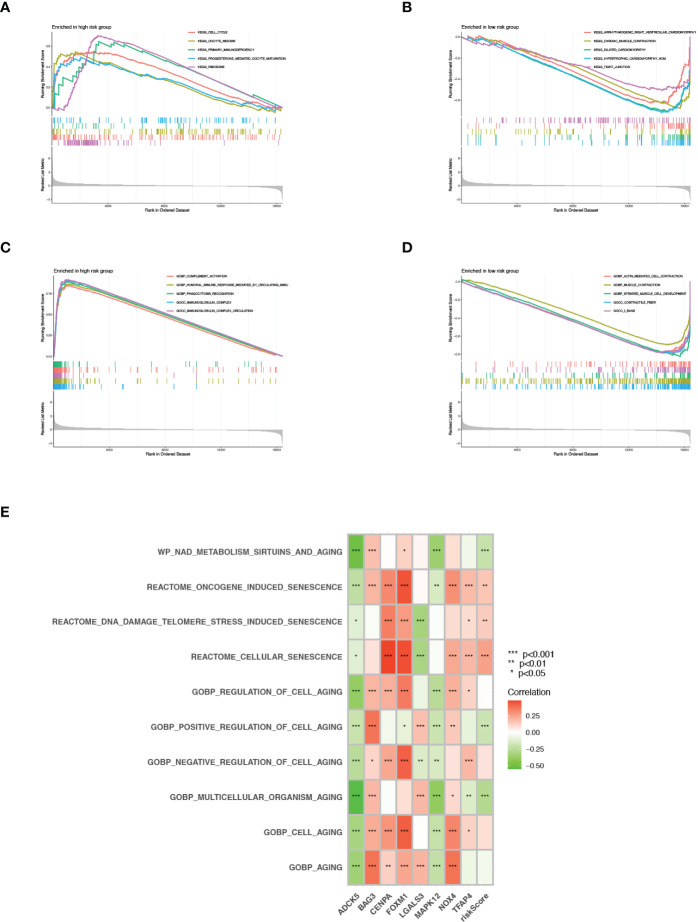
GSEA and GSVA. **(A, B)** KEGG pathway enrichment analysis for high- and low risk groups. **(C, D)** GO functional enrichment analysis for high- and low risk groups. **(E)** GSVA in cell aging related pathways.

Gene set enrichment analysis (GSEA) was conducted to explore further the profiles of signaling pathway activation among two senescence-risk subgroups. As illustrated in **Figures 7A, B**, the enrichment of biological functions in the high-risk group was mainly manifested in the cell cycle, primary immunodeficiency, and the ribosome. In contrast, the top five KEGG pathways in low-risk patients were “arrhythmogenic right ventricular cardiomyopathy”, “cardiac muscle contraction”, “dilated cardiomyopathy”, “hypertrophic cardiomyopathy” and “tight junction”. Based on the GO enrichment analysis, we discovered that the biosignatures in the high-risk patients participated in the positive regulation of these functions, such as “complement activation”, “phagocytosis recognition” and “immunoglobulin complex”. On the contrary, the biological pathways of “muscle contraction” and “contractile fiber” were significantly activated in patients with low-risk scores (**Figures 7C, D**). Furthermore, we verified that risk scores were positively associated with “oncogene-induced senescence”, “telomere stress-induced senescence” and “cellular senescence”, whereas correlating negatively with “the positive regulation of cell aging” and “multicellular organism aging” (**Figure 7E**).

### Immunohistochemical analysis

**Figure 8:**
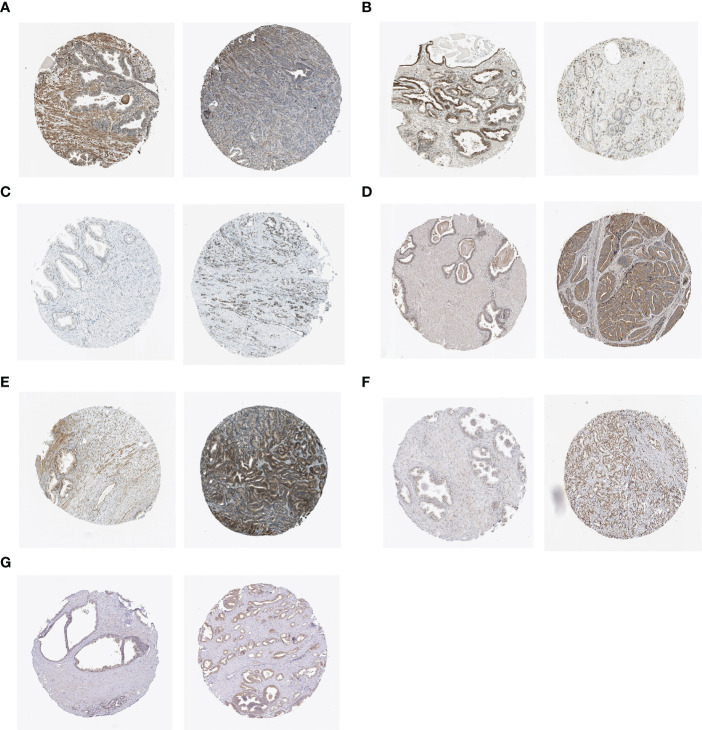
IHC staining of **(A)** BAG3, **(B)** LGALS3, **(C)** CENPA, **(D)** ADCK5, **(E)** FOXM1, **(F)** TFAP4, and **(G)** MAPK12 in normal tissues (left) and PCa tissues (right).

We analyzed the expression of hub genes in the senescence-risk signature using immunohistochemistry (IHC) staining. Compared to the normal tissue, the levels of CENPA, ADCK5, FOXM1, TFAP4, and MAPK were up-regulated in prostate tumor tissue, with a decrease in the expression of LGALS3 and BAG3 (**Figures 8A–G**).

## Discussion

The morbidity of PCa ranks first in urology, accounting for 56% of all urological cancers in 2020 (14). However, diagnosis and therapy against PCa still face enormous challenges on account of enormous inter-tumor heterogeneity regarding clinicopathological, molecular, and morphological characteristics. Therefore, we must tailor appropriate therapeutic strategies to avoid unnecessary treatment for low-grade tumors, while insuring accurate and rapid intervention in high-risk cases.

The role of senescence has attracted considerable attention in a variety of fields. The current perspective indicates that age is the most critical risk factor for prostate tumorigenesis. According to the Hayflick limit, most somatic cells divide naturally up to 40–60 times and eventually undergo cellular senescence, a state that imposes stable cell cycle arrest (15). As an essential biological behavior, senescence works like a safeguard to eliminate abnormal and dysfunctional cells, thereby maintaining the organism’s homeostasis. It has long been deemed as a key barrier against malignant transformation. Cellular senescence is also beneficially implicated in diverse physiological processes, including wound healing, embryogenesis, and inflammation (16). Although senescent cells are present throughout life, their number gradually increases with age.

However, there is increasing evidence that cellular senescence can also be regarded as a component of the tumor phenotype (17). Their excessive accumulation can lead to the commencement and development of age-related chronic illnesses, such as Alzheimer’s disease and tumor formation (18). There remain numerous reports of its adverse effects on the phenotype of the cell or organism. Senescent cells are usually flattened and form giant multinucleated cells (GMCs), where metabolic deregulation, chromatin rearrangement, and resistance to apoptotic stimuli occur (19). Currently, more evidence has linked the tumor progression with the senescent microenvironment, which is mainly attributed to the SASP expression. The SASP refers to a broad spectrum of pro-inflammatory mediators released by senescent cells, including chemokines, MMPs, and angiogenic factors, which remodel the cellular and surrounding environment and affect nearby cells in autocrine and paracrine patterns. SASP has been demonstrated to regulate epithelial-mesenchymal transition (EMT) of tumor cells, thereby increasing their invasiveness (20). In PCa, senescent tumors with PTEN-deficiency evade immune surveillance by intensively triggering immunosuppressive SASP related with recruitment of myeloid-derived suppressor cells (MDSCs) in tumor niche. Previous studies indicated that PCa cells underwent a transient growth arrest upon exposure to either charcoal-stripped serum (CSS) or antiandrogen bicalutamide. Intriguingly, senescent cell populations eventually escaped the growth cessation and turned into castrated-resistant tumors in castrated syngeneic mice. Mechanically, the emergence of CRPC was associated with the generation of androgen receptor splice variants (AR-Vs) mediated by senescence, which further identified senescence as a robust driver of PCa progression (21).

Quantifying the cellular senescence levels for better stratification is still a vital suspending question because of the lack of specific senescence-associated markers (16). In general, current detection means depend on IHC co-staining for several known biomarkers, for example, senescence-associated β-galactosidase activity (SA-β-Gal), p21, and p16^INKA^, to reduce the false positives (22). However, this experimental approach hardly guarantees simultaneous dyeing in certain situations. Additionally, capturing universal senescence features by analyzing transcriptional profiles of senescent cells is gaining increasing attention. However, a suitable tool of senescence quantification in PCa patients remain poorly characterized. Thus, it is urgently needed to develop a computational method of risk stratification for better management.

Nowadays, the revolutionary evolution of bioinformatics vastly facilitates the development of biomedicine, providing great advantages for studying the diagnosis, pathogenesis, and prognosis of diseases. Herein, we first acquired a group of senescence-related genes based on the CellAge database and sifted DEGs between PCa tissues and adjacent normal tissues from the TCGA database. Here, we identified 16 vital SRGs in PCa, eight of which were finally screened out to establish a risk senescence-regulator-gene prognostic model after employing univariate Cox and LASSO regression analyses. The accuracy of the predictive model was further validated using GSE70770 and GSE46602 databases. ROC curve results indicated that the risk model had strong predictive power with respect to BCR. Of note, tracing the clinical features of patients revealed that the high senescence-risk score closely correlated with TNM staging and an adverse outcome. AR signaling pathway was seemingly able to regulate senescence. Surprisingly, supraphysiological androgen levels (SALs) suppressed PCa growth in an AR-dependent manner by inducing cellular senescence, though physiologic levels of androgens boost growth (23). Mechanistically, SAL treatment resulted in an increased level of p16INK4A and p15INK4K, pRb hyperphosphorylation, and inhibition of E2F transcriptional activity (24). Non-genomic AR-AKT- p15INK4K signaling was also involved in androgen-mediated cellular senescence (25). Noteworthily, cellular senescence can be triggered by non-steroidal AR antagonists, such as enzalutamide and bicalutamide (26).

Additionally, we demonstrated the risk model as an independent prognosis-predictive indicator in PCa through performing multivariate Cox regression analysis. Based on the clinicopathological and risk score, we obtained a calibrated nomogram model that achieved a satisfactory validation of the predicted 1-, 3- and 5-year BCR times for PCa. The clinical benefit of immunotherapy varies dramatically among patients, and the response to immunotherapy largely hinges on immunomodulatory factors, such as immune checkpoints, immune cell infiltration, and TMB, which result in tumor heterogeneity (27). The TIDE score was integrated to evaluate the efficiency of immune checkpoint inhibitors (ICIs), and a higher TIDE score correlated with worse ICI response (28). However, high-risk patients exhibited a lower level of TIDE score and may be more sensitive to ICIs. In our signature, the risk score has a significantly positive correlation with TMB defined as the total number of mutations detected per million bases (29). TMB reflects the neoantigen number on the cell surface; therefore, these patients with high TMB levels were likely to respond to immunotherapy. To further determine the relationship between the senescence score and immune status in PCa, we compared the difference in filtration of tumor-associated immune cells among the two risk groups. Patients usually present with increased M2-like macrophage infiltration and decreased T lymphocytes in the high-risk group. The risk score negatively affected the C3 immune subtype that represented the best prognosis. The signature had the tremendous potential to predict drug response for PCa patients. Our study suggested that high-risk patients were more likely to show sensitivity to several conventional anti-PCa agents.

Among the eight essential genes from the signature, the following hub genes deserve to be discussed in depth. The mitogen-activated protein kinase (MAPK) pathway is one of the most extensively studied signaling pathways, and its hype-activation is generally involved in the pathogenesis of human diseases, particularly tumorigenesis (30). Over the past period, the mutations of various genes in this MAPK pathway have been identified, including Raf, Ras, and MEK. These mutational molecules constitutively activate the MAPK signaling to promote the progression of various malignancies. Consistent with other tumors, the MAPK signaling pathway is highly activated in PCa, and its activity is especially associated with androgen independence, therapeutic resistance, and poor prognosis (31). MAPK stabilized the GATA2 protein through suppressing GATA2 ubiquitination/degradation and enhanced its transcriptional expression for AR activation, which resulted in castration resistance (32). As such, MAPK-associated molecules have been regarded as therapeutic targets, and a few attempts have been made to explore the regulatory network of MAPK activation in PCa.

Reactive oxygen species (ROS), critical regulators of redox signaling, are implicated in diverse physiological and pathological processes (e.g., proliferation, metastasis, and differentiation). ROS are both by-products of intracellular metabolism and enzymatic products (33). The NADPH oxidase (NOX) family, a primary source of detectable ROS, contains seven members (Nox1-5 and Duox1-2), catalyze the electron transfer from the cytosolic donor NADPH across biological membranes to generate isoform-specific superoxide and hydrogen peroxide (H_2_O_2_). NOX4-ROS-mediated NF-kB stimulation and subsequent AR expression induced the survival of AR-positive PCa cells (34). Nox4 is unique in the NOX enzymatic family as it is constitutively active. It can mediate oxidative stress/DNA damage, resulting in cellular senescence in a subgroup of prostatic epithelial cells and secondary senescence-associated secretory response (35, 36). Notably, Nox4 induced the biological process of cellular senescence when highly expressed in mouse NIH3T3 fibroblasts *in vitro* (18/33). Recently, several studies showed a significant upregulation of Nox4 expression in PCa patients that experienced BCR following radical prostatectomy and in patients with decreased PCa-specific survival (37, 37).

FOXM1, as a crucial transcription factor, contributes to the phenotype of tumor cells by regulating downstream target genes. After inhibition of FOXM1 expression in PC-3 cells, the downregulated genes were mainly enriched in the DNA repair pathway, specifically in homologous recombination (HR). Additionally, we observed that FOXM1 was aberrantly overexpressed in various human malignancies according to TCGA databases and confirmed prognostic values of its regulatory network (20/44). FOXM1 levels are highly associated with the Gleason score (GS) and acquired resistance in advantage stage of PCa. siRNA-mediated FOXM1 suppression could re-sensitize resistant PCa cells to docetaxel-mediated apoptosis (38). A previous study indicated that FOXM1 could directly bind to PSA promoter/enhancer regions, regardless of the presence of androgen. Hence, FOXM1 may be considered as a novel androgen-independent molecule in CRPC. FOXM1 was also reported to drive the progression of prostate cancer subtype 1 (PCS1), the most aggressive and lethal PCa (39). CENPA is a histone H3-like protein that participates in centromeric nucleosome formation and is recognized as the shared gene between the FOXM1 pathway and PCS (40). Also, CENPA appears positively enriched in the WNT/β-Catenin signaling pathway and acts as the main regulon of Ki-67, a ubiquitous prognostic and proliferative marker widely employed in tumor histopathology (41)(22/22). Herein, we reported that CENPA was up-regulated in PCa tissues, and its overexpression correlated with adverse clinicopathological outcomes in a large cohort (42).

Previous studies revealed that TFAP4 acted as a rate-limiting regulator of adenoma initiation and promoted tumorigenic capability (43). TFAP4 presumably maintains tumor hallmarks by repressing or activating genes that contain CAGCTG elements in their promoter regions, thereby regulating biological processes such as proliferation, epithelial-mesenchymal transition (EMT), and metabolism (44). In prostate carcinoma, TFAP4 was strongly elevated and associated with lymph node metastasis and GS. MiR-22-3p is characterized as a senescence-related microRNA that exerts functions by directly targeting and suppressing SIRT1, CDK6, MDC1, and Sp1 (45, 46). However, TFAP4-deficient tumor cells displayed increased spontaneous DNA damage, chromosomal instability (CIN), and cellular senescence mediated by direct or indirect MiR-22-3p repression by AP4.

There were some limitations and deficiencies in this present study. First, eight hub genes were selected to construct the prognosis-predictive model, which may increase healthcare costs for each patient. Hence, our signature needs further improvement and simplification. Second, the treatment modalities (surgery, radiotherapy, and ADT) in patients were not taken into consideration, which may cause certain inaccuracy problems. Third, experiments *in vivo* and *in vitro* should be performed to understand the biological functions of the eight SRGs; meanwhile, the prognostic value of the signature needed to be further validated clinically.

In conclusion, we constructed and validated a practical senescence-risk algorithm based on eight SRGs, and the signature hopefully acted as a potential risk model for PCa-prognostic prediction. Additionally, the research discovered new information on the correlation relationship between the SRG-score with TMB, immune microenvironment, and drug sensitivity, which can provide vital insight for tailoring therapeutic choices for PCa patients.

## Data availability statement

The datasets presented in this study can be found in online repositories. The names of the repository/repositories and accession number(s) can be found in the article/**Supplementary Material**.

## Author contributions

CH wrote the original draft; YD, BY, and PH performed data collection. BH and TW performed the supervision. JL and QX were responsible for project design. QX and XL revised the manuscript critically and approved the final version. XL provided funding support. All authors contributed to the article and approved the submitted version.

## Supplementary material

Supplementary Figure 1
**(A)** Correlation among the 8 genes. **(B)** Risk scores of each patient in the TCGA cohort. **(C, D)** PCA based on 8 selected SRGs and all SRGs in high- and low-risk patients. **(E)** Relationship of clinical characteristics with the expression of genes enrolled in this signature.

Supplementary Figure 2
**(A, B)** Distribution of risk score in the GSE70770 and GSE46602 test database. **(C, D)** BCR status of each patient in the two cohort.
