# Supplementary figures and images for: Identification of a novel senescence-associated signature to predict biochemical recurrence and immune microenvironment for prostate cancer
Source: Front Immunol. 2023 Feb 20;14:1126902. doi: 10.3389/fimmu.2023.1126902 (PMC9986540; doi:10.3389/fimmu.2023.1126902)

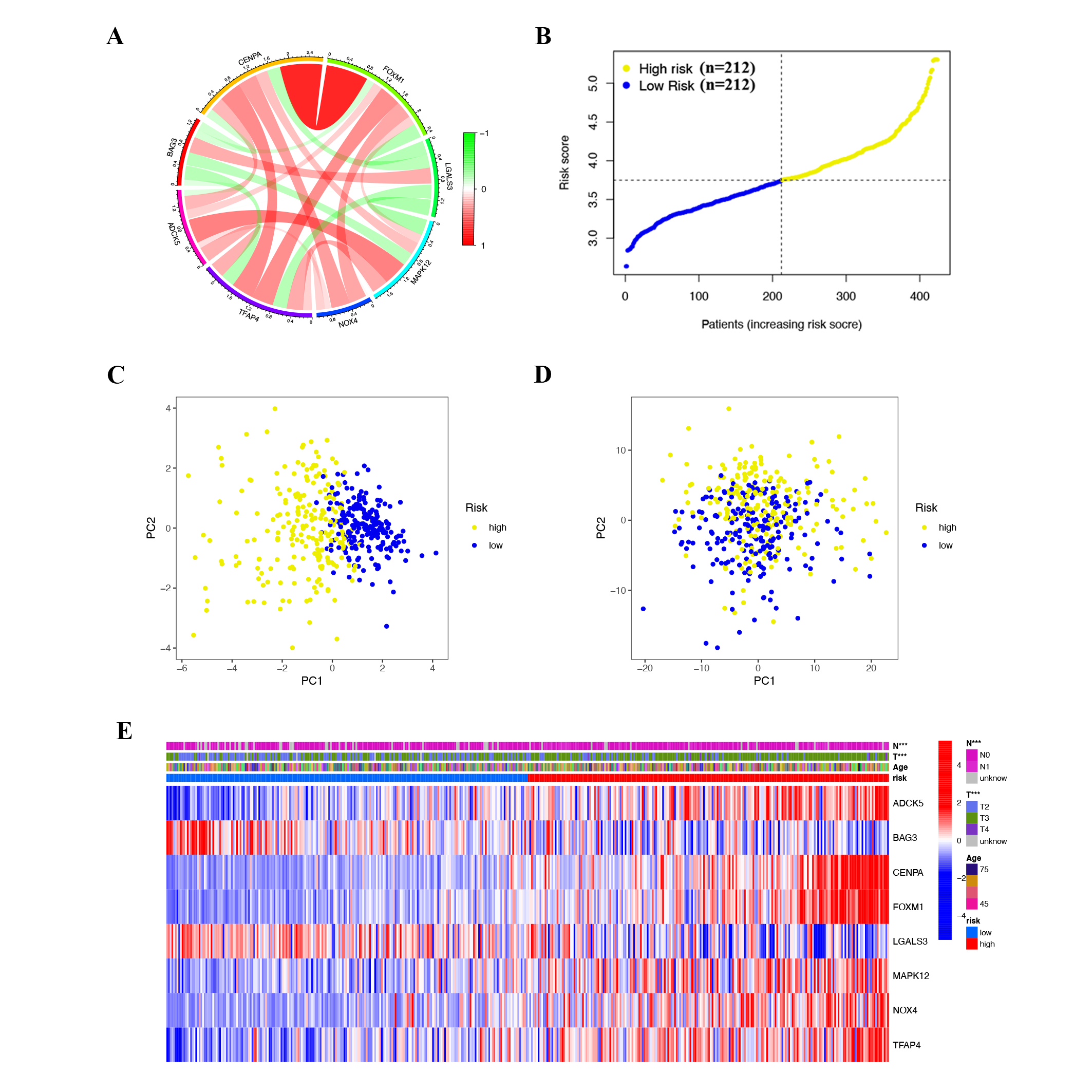

Supplement: Supplementary Figure 1 — (A) Correlation among the 8 genes. (B) Risk scores of each patient in the TCGA cohort. (C, D) PCA based on 8 selected SRGs and all SRGs in high- and low-risk patients. (E) Relationship of clinical characteristics with the expression of genes enrolled in this signature. [file Image_1.tif]

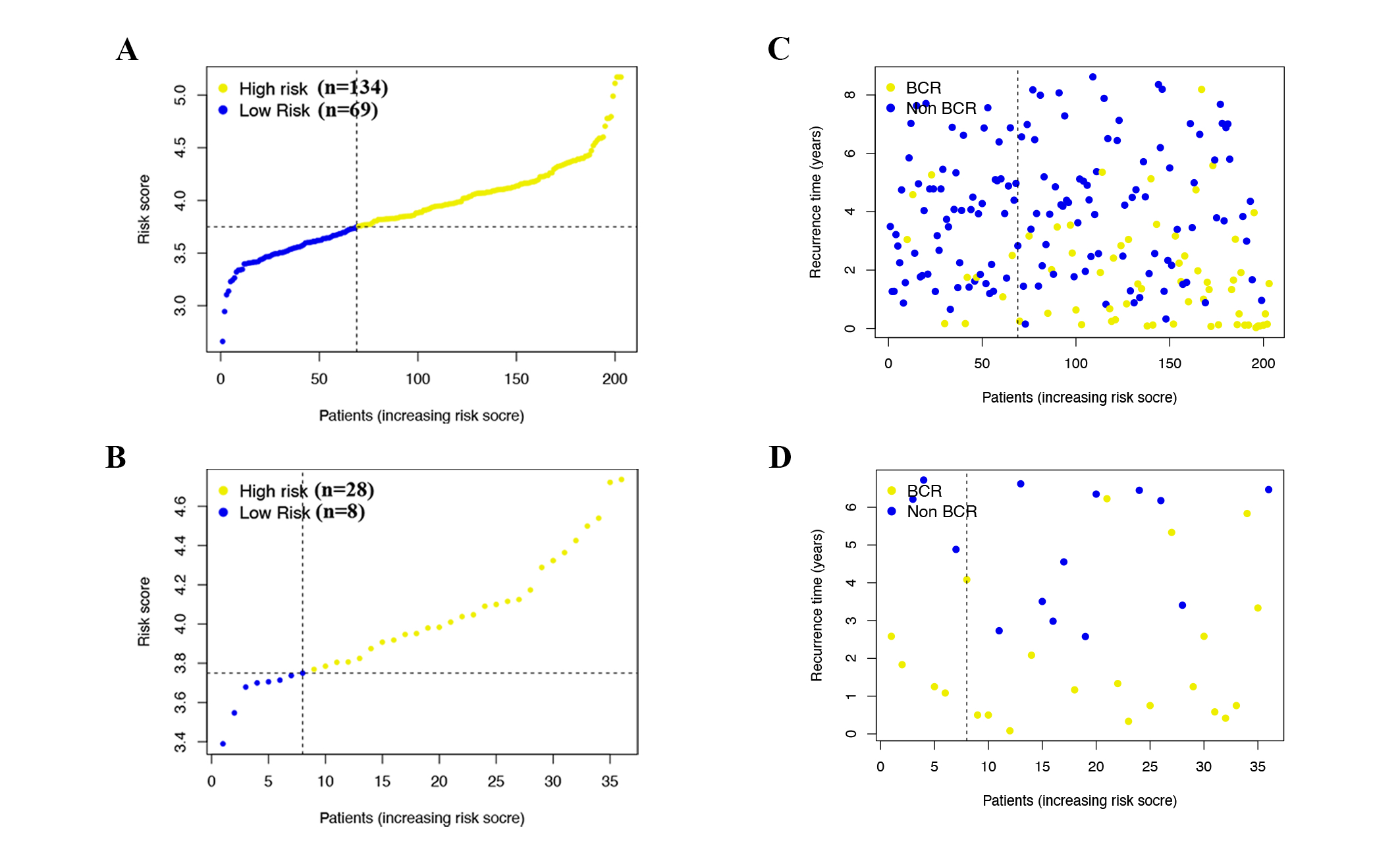

Supplement: Supplementary Figure 2 — (A, B) Distribution of risk score in the GSE70770 and GSE46602 test database. (C, D) BCR status of each patient in the two cohort. [file Image_2.tif]
